# Clinical Implementation of a 6D Treatment Chair for Fixed Ion Beam Lines

**DOI:** 10.3389/fonc.2021.694749

**Published:** 2021-06-23

**Authors:** Jiayao Sun, Lin Kong, Zhi Chen, Dan You, Jingfang Mao, Xiyin Guan, Xiaodong Wu, Yinxiangzi Sheng

**Affiliations:** ^1^ Department of Medical Physics, Shanghai Proton and Heavy Ion Center, Shanghai, China; ^2^ Shanghai Key Laboratory of Radiation Oncology (20dz2261000), Shanghai, China; ^3^ Shanghai Engineering Research Center of Proton and Heavy Ion Radiation Therapy, Shanghai, China; ^4^ Department of Radiation Oncology, Shanghai Proton and Heavy Ion Center, Fudan University Cancer Hospital, Shanghai, China; ^5^ Department of Medical Physics, Shanghai Proton and Heavy Ion Center, Fudan University Cancer Hospital, Shanghai, China; ^6^ Department of Radiation Oncology, Shanghai Proton and Heavy Ion Center, Shanghai, China

**Keywords:** carbon-ion radiotherapy (C-ion RT), treatment chair, upright posture, intra-fraction movement, setup error

## Abstract

**Purpose:**

To verify the practicality and safety of a treatment chair with six degrees of freedom (6DTC) through demonstrating the efficacy of the workflow in clinical settings and analyzing the obtained technical data, including intra-fraction patient movement during the use of the 6DTC.

**Materials and Methods:**

A clinical study was designed and conducted to test the clinical treatment workflow and the safety of the 6DTC. Based on the demonstrated dosimetric advantages, fifteen patients with head and neck tumors were selected and treated with the 6DTC. The positional error at the first beam position (PE-B1) and the second beam position (PE-B2) were analyzed and compared with the results from daily quality assurance (QA) procedures of the 6DTC and imaging system performed each day before clinical treatment. The intra-fraction patient movement was derived from the total patient alignment positional error and the QA data based on a Gaussian distribution formulism.

**Results:**

The QA results showed sub-millimeter mechanical accuracy of the 6DTC over the course of the clinical study. For 150 patient treatment fractions, the mean deviations between PE-B1 and PE-B2 were 0.13mm (SD 0.88mm), 0.25mm (SD 1.17mm), -0.57mm (SD 0.85mm), 0.02° (SD 0.35°), 0.00° (SD 0.37°), and -0.02° (SD 0.37°) in the x, y, z (translational), and u, v, w (rotational) directions, respectively. The calculated intra-fraction patient movement was -0.08mm (SD 0.56mm), 0.71mm (SD 1.12mm), -0.52mm (SD 0.84mm), 0.10° (SD 0.32°), 0.09° (SD 0.36°), and -0.04° (SD 0.36°) in the x, y, z, u, v, w directions, respectively.

**Conclusions:**

The performance stability of the 6DTC was satisfactory. The position accuracy and intra-fraction patient movement in an upright posture with the 6DTC were verified and found adequate for clinical implementation.

## Introduction

Radiation therapy with proton or carbon-ion beams offers physical and biological advantages over x ray beams for many clinical indications (1, 2). An optimal ion beam plan can often be achieved with only two to four beam entry angles; either by rotating the beam delivery gantry, rotating the patient positioning table, or both. Selection of the angles is crucial for achieving the desired target dose coverage while minimizing the dose to the organs at risk (OARs). Most operating proton centers are equipped with one or more rotating gantries. Due to the high cost of carbon-ion beam gantries, however, carbon ion centers typically only have fixed direction beam lines (3). The flexibility of beam orientation for achieving optimal plans and treatments is naturally compromised when using fixed beam lines compared to rotating gantries. To overcome this disadvantage, a treatment chair with six degrees-of-freedom (6DTC) was designed, manufactured, and installed in Shanghai Proton and Heavy Ion Center (SPHIC) for use with a fixed beam line. The 6DTC consists of a 360°-rotating platform, a six-degree-of-freedom (6DOF) hexapod platform, an XYZ-translation platform, and a seat with an adjustable height carbon fiber head and shoulder fixation interface plate (4). Before the clinical study with patients commenced, a series of measurements to test the performance of the 6DTC showed that it had met the requirements for clinical applications (5).

Due to the high geometrical and physical definition of ion beams, patient localization and position correction are essential. This is especially so for the treatment of head and neck cancers given the complexity of the anatomy and the proximity of tumors to critical organs (6, 7). In most modern ion beam therapy facilities, image-guided radiotherapy (IGRT) systems with orthogonal kV images or cone beam CT (CBCT) are used to obtain position correction vectors for patient alignment. Treatments with patients in a lying posture are most commonly used in radiation therapy and there have been many reports of that clinical experience including setup accuracy and intra-fraction patient movement (8–13). On the other hand, there have been only a few studies that reported on the experience of radiation treatments using an upright posture, either with x ray or ion beams (14–21). To the best of our knowledge, beside a preliminary study of intra-faction movement in seated posture by films in 1980s (22), neither the clinical workflow nor the intra-fraction patient movement of the treatment with patients in an upright posture on a chair has been reported after the advent of image guidance.

A clinical trial to verify the practicality and safety of the 6DTC was designed and conducted at SPHIC. The results presented herein demonstrate the efficacy of the workflow for the clinical implementation of the 6DTC. Furthermore, the obtained technical data, including the intra-fraction patient movement, have not only confirmed the adequate accuracy of the 6DTC for clinical applications, but also provided the direction for future improvements of the 6DTC.

## Materials and Methods

For the clinical implementation of the 6DTC, two sets of policies and procedures were developed: first for technical quality assurance (QA) to assure the accuracy and mechanical performance of the chair, and second for the clinical workflow from simulation through planning, patient alignment, and treatment delivery.

### Daily QA of the 6DTC With Rigid Phantom

An anthropomorphic head phantom (PBU-60, KYOTO KAGAKU, Japan) was used for performing the daily QA of the 6DTC before clinical treatment every day. The daily QA procedure was described in detail previously (5). In brief, a random setup error was manually introduced when positioning the head phantom on the chair at the pre-treatment position which was also the setup position. The positional error at the pre-treatment position (PE-P) was obtained by comparing a first pair of orthogonal kV x-ray images with digitally reconstructed radiographs (DRRs) generated from the planning CT images. The 6DTC was subsequently rotated to the treatment position as provided in the treatment plan without applying the correction vector of PE-P, followed by a second pair of KV images to obtain the positional error at the treatment position (PE-T). The correction vector of PE-T was applied, and a third pair of orthogonal kV x-ray images were acquired to obtain the residual positional error at the treatment position (RPE-T).

In essence, the daily QA executes a setup simulation with a head phantom. The purpose of the third pair of orthogonal kV x-ray images is to confirm the final alignment accuracy after the corrections have been applied. The residual positional errors determine whether the performance integrity of the chair is acceptable to proceed with its clinical usage each day.

In this paper, the daily QA data was acquired on each of the 91 treatment days over a period of nine months were recorded. The deviations between PE-P and PE-T and between PE-T and RPE-T were analyzed to assess the performance stability of the chair.

### Clinical Implementation

**Figure 1:**
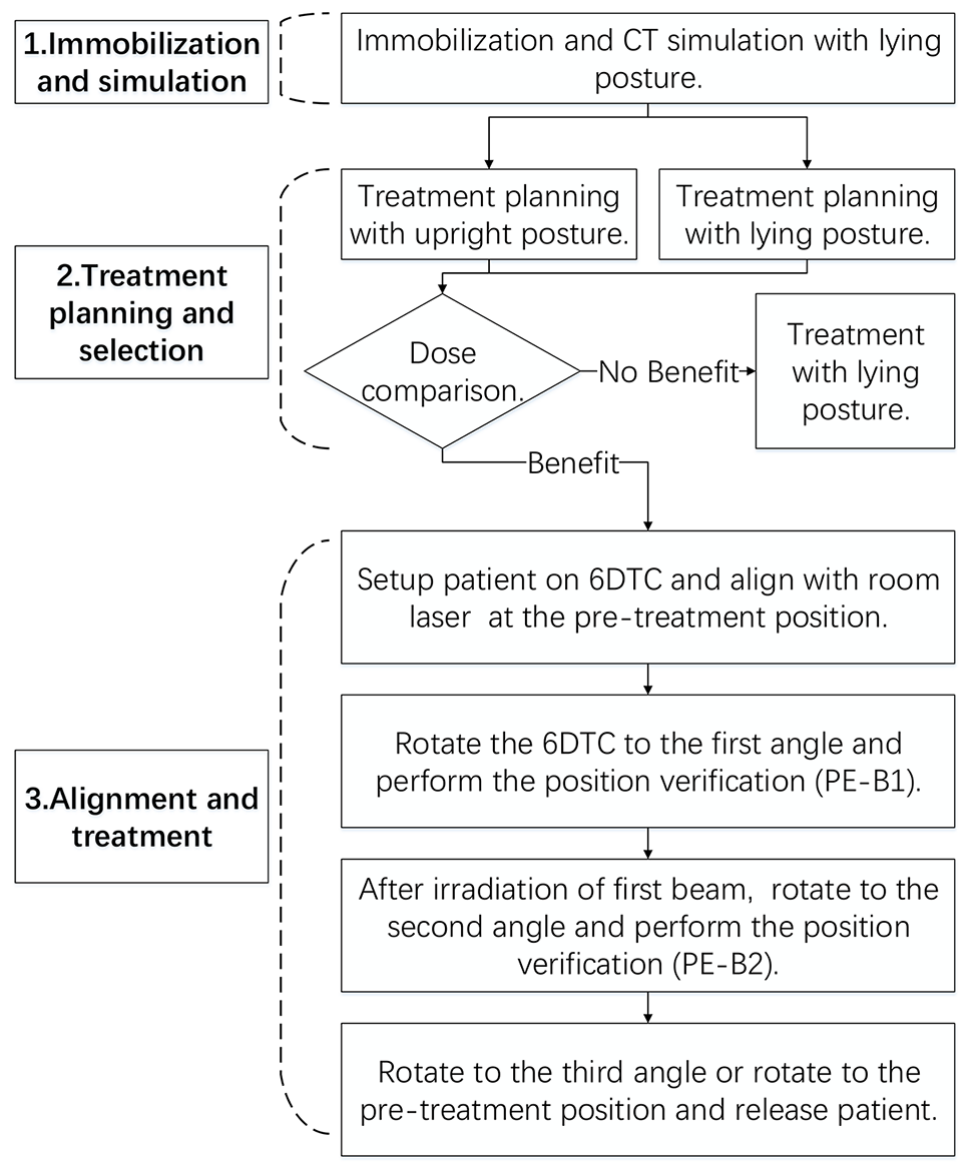
The clinical treatment workflow for this clinical trial.

A clinical study was designed to evaluate the feasibility of the clinical implementation of the 6DTC. The structure of the study followed the 6DTC clinical treatment workflow as shown in **Figure 1**. The implementation of the 6DTC involves three basic steps described in the following subsections.

#### Immobilization and CT Simulation

Due to the design of the fixation equipment, the patient immobilization procedure was the same regardless of whether the posture during treatment would be lying or sitting. All patients were scanned in the lying position (23, 24). A low-density foam cradle attached to a head and shoulder fixation interface plate was used to immobilize each patient’s head, neck, and shoulders with a nine-pin commercial thermoplastic face mask. Planning CTs were acquired using a 1.5 mm slice thickness in helical mode and then transferred to the Syngo^®^ treatment planning system (TPS) (V13B, Siemens, Germany). When the positioner type of “chair” was selected, the CT image set was re-orientated automatically by the TPS to change the patient posture from lying to sitting. The patient’s internal anatomy was assumed to remain rigid during the re-orientation.

#### Treatment Planning and Selection of Treatment Technique

Two types of treatment plans were generated for each patient; one using the treatment table and one using the chair. The planning objectives were to cover at least 95% of clinical target volume (CTV) with 95% of the prescription dose and to minimize the dose to the OARs using two to three beam entry angles. Since the lack of robust planning technique in Syngo, planning target volume (PTV) was added depending on individual factor such as beam angle chosen and was ranged from 3-5mm (23, 25). CTV coverage and OARs dose were compared for the two types of plans. Patients were selected for the treatment with 6DTC only if the chair plan fulfilled three eligibility criteria: superiority in sparing the OARs, comparable target coverage, and without increasing the penetration uncertainty. Patients with CTV lower than the head were excluded.

#### Alignment and Treatment

**Figure 2:**
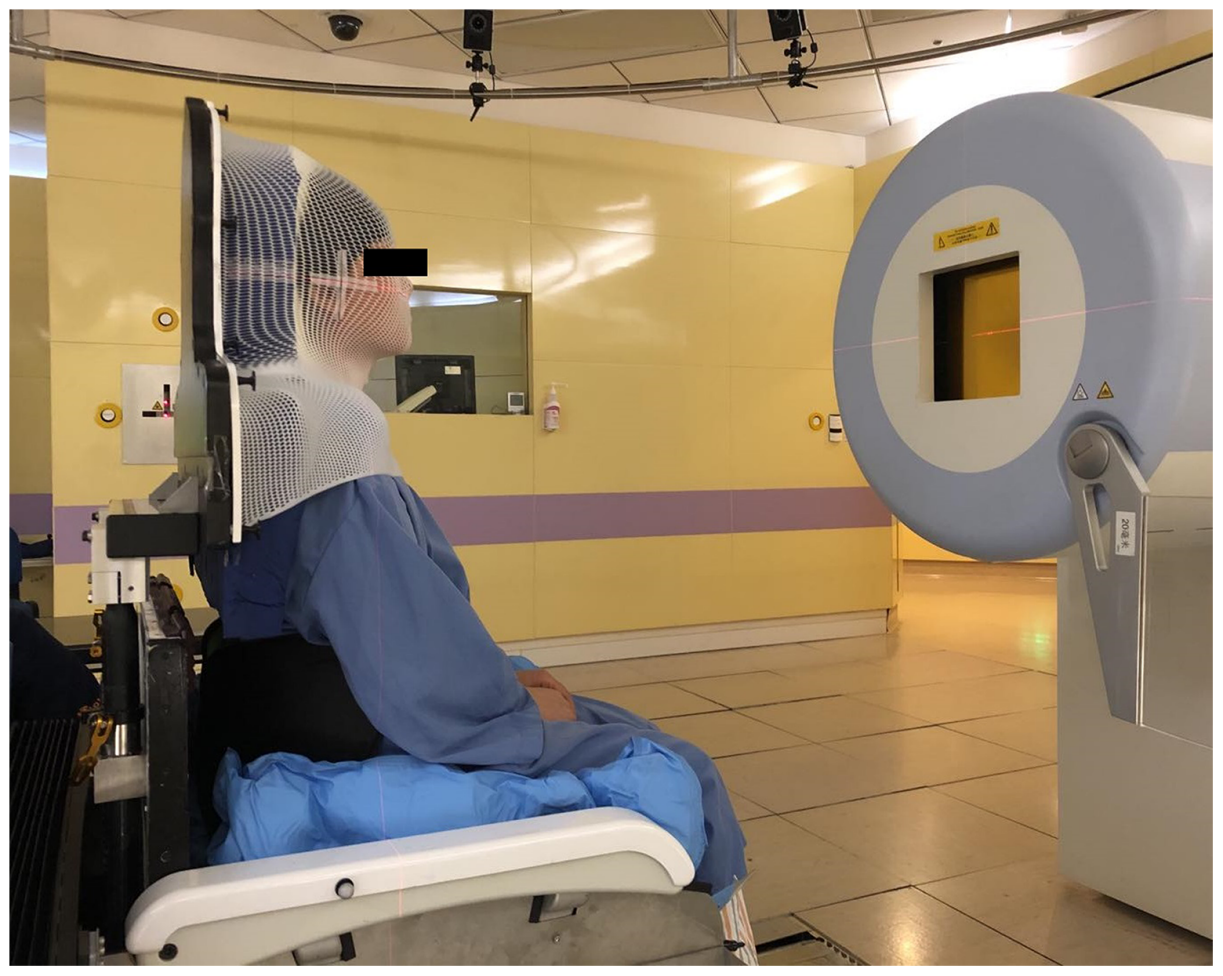
The patient setup on the 6DTC.

For each fraction of treatment, the patient alignment was carried out as follows:

A Vac-lok cushion (CIVCO Radiotherapy, USA) was used to register the patient’s thigh and butt and a back cushion was used to support the back when setting up the patient on the chair (**Figure 2**). The patient’s head was immobilized with the customized thermoplastic mask and foam cradle. The patient was initially aligned with the room lasers at the pre-treatment position (with the 6DTC facing the nozzle), then rotated to the planned chair iso rotation angle of the first beam. A pair of orthogonal kV x-ray images were acquired and the registration of the kV images with the DRRs was performed with respect to the bony anatomy. The positional errors at the first beam position (PE-B1) (three translational shifts lateral x_B1_, longitudinal y_B1_, vertical z_B1_, and three rotational shift iso u_B1_, pitch v_B1_, roll w_B1_) were then recorded. The coordinate system follows the International Electrotechnical Commission (IEC) convention and was well illustrated by Sheng et al. (5) The patient position was then corrected by applying the PE-B1 to the 6DTC after which the planned beam was delivered. After finishing the irradiation of the first beam, the 6DTC was rotated to the planned chair iso rotation angle for the second beam. The second pair of orthogonal KV x-ray images were acquired and registered with the DRRs to obtain the positional errors for the second beam position (PE-B2) (three translational shifts x_B2_, y_B2_, z_B2_, and three rotational shift u_B2_, v_B2_, w_B2_). The 6DTC was then repositioned and the second beam delivered. The last procedure was repeated if there were more than two beams for the fraction of treatment. The treating physician was required to be present to review and approve the alignment procedures for each fraction of the treatment.

### Analysis of Position Accuracy

In a previous report, it was shown that under x-ray-based image guidance, the 6DTC could provide positional alignment with sub-millimeter accuracy with a rigid phantom in an upright posture (5). The sources of errors came from different components of the chair and the imaging system. When applied to clinical settings, however, an additional source of positioning error is present due to patient motion. This source should also be assessed and addressed.

In this clinical study, the chair and imaging related mechanical errors and the patient-specific intra-fraction position error were derived and evaluated. The measurements representing the composite effects from both sources of errors were identified, collected and analyzed. Specifically, the deviation between PE-T and PE-P representing the positional errors introduced by both the chair and the IGRT system (5) can be considered as a Gaussian distribution denoted as $N_{1}\left(\mu_{1},\sigma_{1}^{2}\right)$. In this study, $N_{1}\left(\mu_{1},\sigma_{1}^{2}\right)$ was calculated from the daily QA data with the rigid phantom. In this study, the intra-fraction movement from patients was not directly assessed; rather, the net excursion from the final setup position of the first beam to the final setup position of the second beam was used as a presentation of patient-specific intra-fraction movement; this excursion can be considered as another independent Gaussian distribution, denoted as $N_{2}\left(\mu_{2},\sigma_{2}^{2}\right)$. In the afore-described alignment procedure, the deviation between PE-B1 and PE-B2 was the composite or total positional errors introduced by the chair/IGRT system and the patient’s intra-fraction movement was denoted as $N_{3}\left(\mu_{3},\sigma_{3}^{2}\right)$. It naturally follows that

(1) $$N_{3}\left(\mu_{3},\sigma_{3}{\;}^{2}\right)=N_{1}\left(\mu_{1},\sigma_{1}{\;}^{2}\right)+N_{2}\left(\mu_{2},\sigma_{2}{\;}^{2}\right)$$

(2) $$\mu_{3}=\mu_{1}+\mu_{2}$$

(3) $$\sigma_{3}{\;}^{2}=\sigma_{1}{\;}^{2}+\sigma_{2}{\;}^{2}$$

where *μ_i_* is the expectation of Gaussian distribution, and *σ_i_* is the standard deviation.

The study was reviewed and approved by the Institutional Review Board (Approval No: 1812-29-04).

## Results

### Patient Accrual

15 patients were selected to receive treatments with the 6DTC based on the criteria mentioned above. The characteristics of these 15 patients are listed in **Table 1**.

**Table 1:** Characteristics of patients.

Characteristics	No. of patients
**Age**	
Median (Range)	48 (21-83)
**Weight (kg)**	
Median (Range)	61 (49-105)
**Height (cm)**	
Median (Range)	168 (146-177)
**Gender**	
Male	9
Female	6
**KPS**	
100	7
90	8
**Disease (Tumor Site)**	
Nasopharyngeal carcinoma (NPC) (Nasopharyngeal)	8
Meningioma (Cerebellopontine Angle)	1
Adenoid cystic carcinoma (Maxillary sinus)	1
Squamous cell carcinoma (External auditory canal)	1
Adipose-derived tumor (Orbit)	1
Osteosarcoma (Orbit)	1
Atypical carcinoid tumor (Slope)	1
Chordoma (Skull base)	1
**No. of fractions treated with 6DTC**	
5*	9
8	1
10	1
20	3
27	1
**No. of beams per plan**	
2	14
3	1

*For these nine patients, the prescriptions were 56 Gy(RBE) delivered by either protons or x rays in 28 fractions using a lying posture plus 17.5Gy(RBE) by carbon ions in five fractions using a sitting posture.

### Treatment Planning, Dosimetric Comparison and Selection of Treatment Technique

For each of the fifteen patients, the target received comparable coverages with the chair and table plans. The volumes of the CTV that received ≥95% of the prescription dose (V95) were 98.81% ± 1.45% (mean ± standard deviation) and 98.48% ± 2.14% for the chair plans and table plans respectively, i.e., no significant difference (*p*=0.334).

For the OARs, in the eight NPC patients, chair plans achieved a lower mean dose in both the bilateral parotids and cochleae. Specifically, the mean doses to the right parotid, left parotid, right cochlea and left cochlea were 28.5% ± 18.3%, 28.0 ± 21.0%, 26.6% ± 22.7%, and 32.9% ± 25.0% lower with the chair plans comparing with the table plans, respectively. (*p*=0.006, 0.002, 0.017, and 0.016, respectively.) Furthermore, chair plans could also decrease the mean dose to temporal lobes by 49% ± 27%, compared with table plans. (*p*=0.007)

In the two patients with tumors around the orbits, chair plans showed 50% to 100% lower mean dose to the contralateral eye globe. For the other five patients, the chair plans reduced the mean dose of the parotid and the cochlea by 4% to 26%.

### Treatment Delivery

#### Daily QA of 6DTC With Rigid Phantom

On each day when at least one fraction of a chair treatment was scheduled, the daily QA of the 6DTC with a rigid phantom was performed.

The mean deviations between PE-T and PE-P were 0.21mm (SD 0.70mm), -0.49mm (SD 0.36mm), -0.02mm (SD 0.11mm), -0.08 (SD 0.13°), -0.09(SD 0.09°), and 0.02 (SD 0.08°) in the x, y, z, u, v, w directions, respectively.

The mean deviations between RPE-T and PE-T were 0.18mm (SD 0.23mm), -0.18mm (SD 0.18mm), 0.06mm (SD 0.12mm), 0.09° (SD 0.13°), 0.02°(SD 0.14°), and 0.01° (SD 0.09°) in the x, y, z, u, v, w directions, respectively.

The acceptance criteria for the deviation between PE-T and PE-P and between RPE-T and PE-T is within 1.5mm and 1.5°. If the deviation is out of tolerance, the daily QA must be repeated, and if the result still fails, the chair would need to be re-calibrated, especially for the rotation center of the 360°-rotating platform. Having performed the daily QA for the 91 fractions of treatments, only two of them failed, which were promptly restored after re-calibrating the rotation center.

#### Patient Alignment

**Figure 3:**
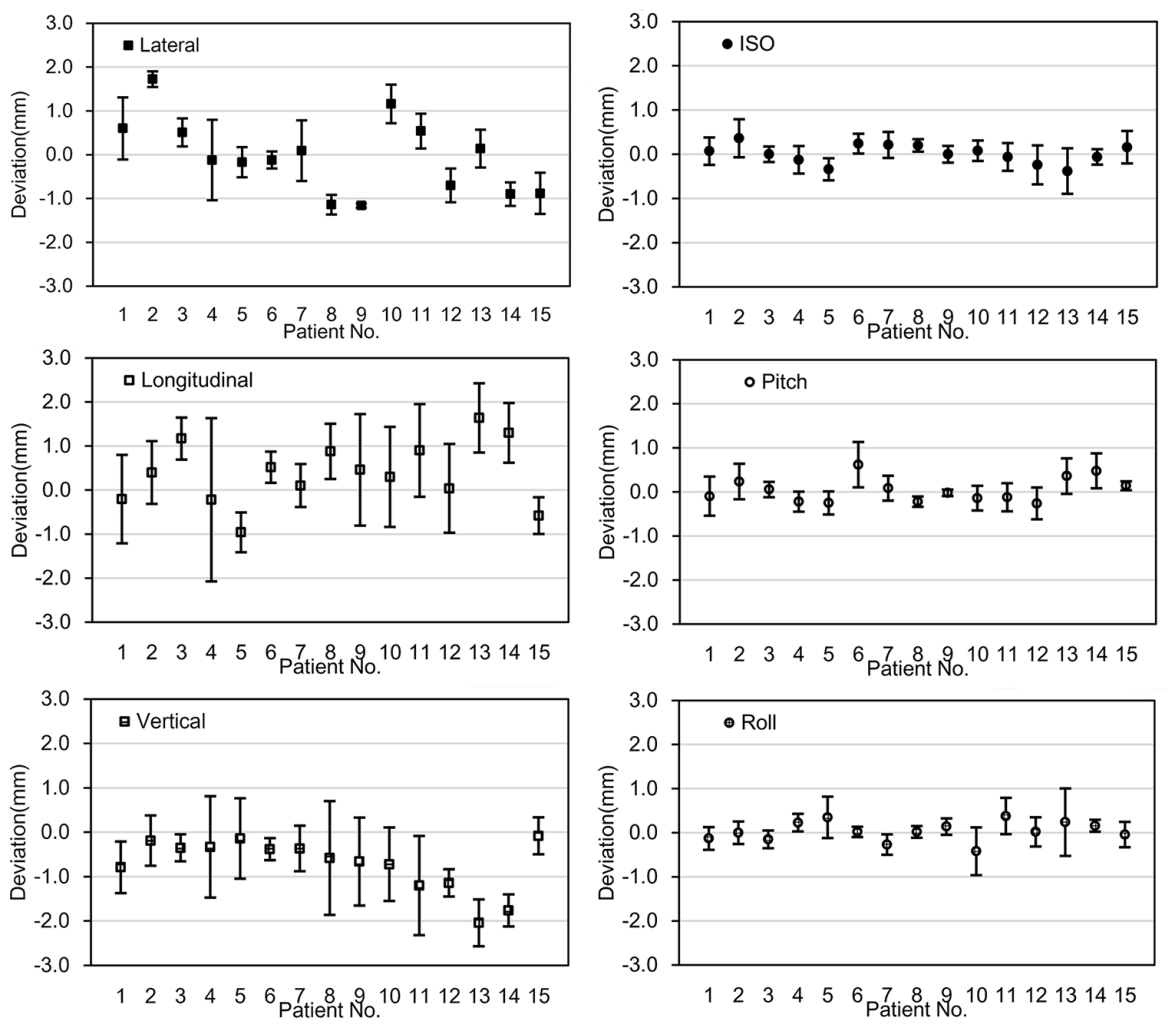
The deviation between PE-B1 and PE-B2. The x-axis represents the fifteen individual patients; the y-axis represents the mean value of the deviation of the specific patient in 6 degrees of freedom. Error bars represent for the standard deviation.

For the fifteen patients selected for chair-treatment, a total of 150 fractions of treatments were delivered, with fourteen patients having two beam angles and one patient having three beam angles in each treatment fraction. The average treatment time from the patient sitting down on the chair to leaving the chair was 30 minutes (SD 7 minutes). The PE-B1 and PE-B2 were recorded and their deviations, defined as the total positional error were calculated. **Figure 3** shows the mean deviations in all six degrees of freedom for each patient.

The mean deviations between PE-B1 and PE-B2 for all patients were 0.13mm (SD 0.88mm), 0.25mm (SD 1.17mm), -0.57mm (SD 0.85mm), 0.02° (SD 0.35°), 0.00° (SD 0.37°), and -0.02° (SD 0.37°) in the x, y, z, u, v, w directions, respectively. Of the mean translational deviations between beams, 91.1% were within ±1.5mm, while all the mean deviations were within ±2mm except for the vertical value of one patient (No. 13). For rotational deviations, only one value exceeded 0.5°.

For each fraction, as shown in **Table 2**, the frequencies of deviation greater than 1 mm in the x, y, z, directions were 27.3%, 33.3%, and 26.7%, respectively. The frequencies of deviation greater than 1° in the u, v, w directions were 1.3%, 2.7%, and 2.0%, respectively. There were no deviations >4mm in the x and z translational directions and only 2 in the y direction. There were no deviations > 2° in any of the rotational directions. Note that these deviations were those present before the position correction was applied to the alignment for the second angle.

**Table 2:** Frequency of deviation [%] between PE-B1 and PE-B2 by thresholds.

	Translational				Rotational		
	x	y	z		u	v	w
>1mm	41(27.3)^*^	50(33.3)	40(26.7)	>1°	2(1.3)	4(2.7)	3(2.0)
>2mm	1(0.7)	10(6.7)	8(5.3)	>2°	0(0.0)	0(0.0)	0(0.0)
>3mm	0(0.0)	2(1.3)	1(0.7)	>3°	0(0.0)	0(0.0)	0(0.0)
>4mm	0(0.0)	2(1.3)	0(0.0)	>4°	0(0.0)	0(0.0)	0(0.0)
>5mm	0(0.0)	0(0.0)	0(0.0)	>5°	0(0.0)	0(0.0)	0(0.0)

^*^Data in parentheses are percentages.

#### Intra-Fraction Movement

**Figure 4:**
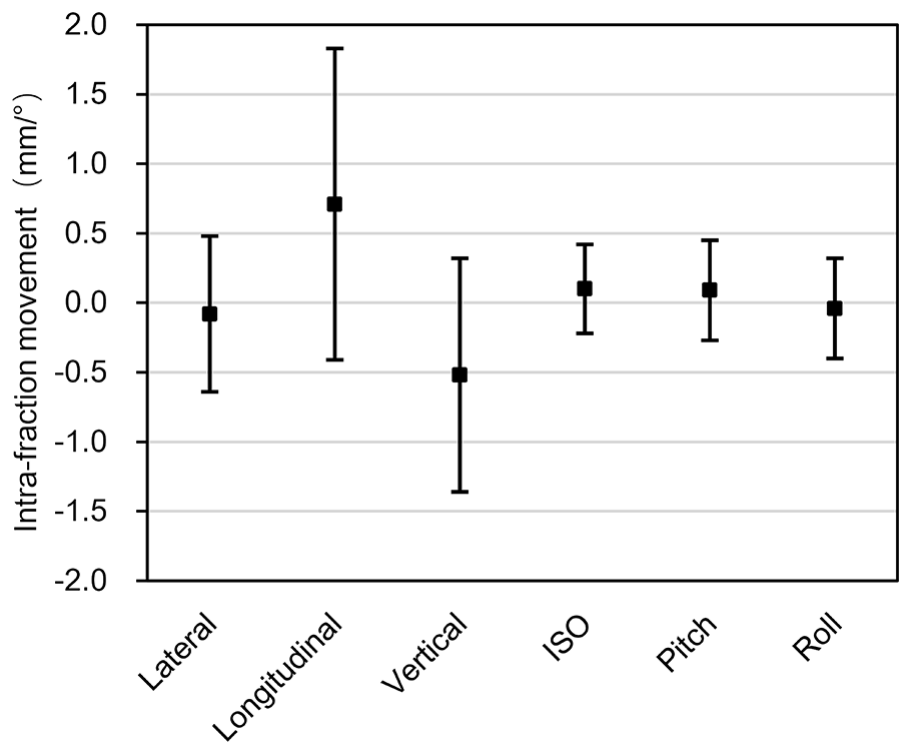
Intra-fraction patient movements. The x-axis represents the three translational directions and three rotational directions, while the y-axis represents the amplitude of the intra-fraction patient movements. The error bars represent the standard deviation.

Based on the formula 1-3, the mean intra-fraction patient movements were calculated, which were -0.08mm (SD 0.56mm), 0.71mm (SD 1.12mm), -0.52mm (SD 0.84mm), 0.10° (SD 0.32°), 0.09° (SD 0.36°), and -0.04° (SD 0.36°) in the x, y, z, u, v, w directions, respectively (**Figure 4**).

## Discussion

We have introduced a clinical treatment workflow for using the 6DTC to deliver particle treatment in an upright seated posture and presented our experience in a feasibility clinical study. During the course of this clinical study, a total of 320 head and neck patients received particle radiotherapy with the table and the 6DTC. Of these patients, 15 patients were selected to receive the treatment of the 6DTC. For other patients, due to no obvious benefit from the 6DTC, lying posture was chosen. Using our designed workflow, the immobilization procedures for upright treatment were designed to be the same as the lying treatment. If the advantage of the chair plan was observed, treatment in the upright posture can be easily achieved by transferring the alpha-cradle (with the bottom cut off) to the chair head/shoulder fixation interface plate. On the other hand, if no benefit was indicated, patients can go on to receive the treatment in lying posture without changing the immobilization device or undergoing planning CT simulation again.

The rotating gantries of particle therapy provide a wide range of beam entry orientations, and are therefore desirable for obtaining optimal plans and treatments. Notwithstanding, there are challenging issues, including inaccuracy of the iso-center due to its excessive weight and the instability of beam qualities when the beam nozzle rotates. Moyers et al. (26) reported the maximum iso-center shifts of 1.17 mm along the clockwise path and 1.26 mm along the counterclockwise path for proton rotating gantries. Kato et al. (27) also reported up to 1mm of deviation at iso-center in proton rotating gantries. With a markedly more massive carbon-ion gantry, one could only expect the worse, although no data have been reported. The initial intent of developing the 6DTC was to compensate for the lack of beam rotation with a fixed beam line. Our studies have shown that the mechanical accuracy of the 6DTC is comparable to the rotating gantries. Given its simplicity over the gantry, the improvement to further reduce errors will be logically easier to achieve with the chair. With the development of vertical CT (28), when equipped with a 6DTC, the fixed beam nozzle may become the preferred configuration for modern carbon-ion radiotherapy facilities.

Positional shifts of patients related totable rotation during treatments have been reported in several studies. Rosenfelder et al. (29) reported up to -0.8 ± 0.7 mm positional shifts in the longitudinal direction for noncoplanar beams in external beam radiotherapy. Sarkar et al. (30) reported an average shift of 0.6 ± 0.9mm in the lateral direction for noncoplanar beams in stereotactic radiotherapy (SRT) and stereotactic radiosurgery (SRS) using frameless setup. Lewis et al. (31) reported an average shift of 0.55 ± 0.43 mm in the lateral direction for noncoplanar beams in SRS. Overall, the shifts observed in this study are comparable to those found in these other studies. The position deviation between PE-B1 and PE-B2 is attributed to the positional errors introduced by both the chair and the patients’ intra-fraction patient movement. The maximum mean deviation in our study was -0.57 ± 0.85mm (z-direction). The maximum combined translational and rotational shifts reported by Sarkar et al. (30) were -3.1mm and 4.2°, while we found 4.6mm and 1.6° in our study. By analyzing the CBCT performed before and after IMRT treatment Den et al. (32) reported the residual error frequencies ranging from 23.0% to 34.0% and 3.0% to 5.4% for >1mm and >3mm thresholds, while Lu et al. (33) reported frequencies from 17.5% to 30.8% and from 0.0% to 4.5% for 1mm and 2mm thresholds using similar methods. In our study, the frequencies of the deviations between PE-B1 and PE-B2 with thresholds >1mm, >2mm and >3mm ranged from 26.7% to 33.3%, 0.7% to 6.7%, and 0.0% to 1.3%, respectively, again, comparable to the published data. For the frequencies of the rotational deviation, 1.3% to 2.7% exceeded 1° threshold, while none exceeded 2°.

Even with an immobilizing thermoplastic mask, the considerable patient movement had been reported for treatments with lying posture (13). For patients treated in sitting posture for about 30 minutes (including setup, position correction procedures, and treatment delivery), the potential impact of intra-fraction patient movement should call for the same if not more attention. The results of the sub-millimeter magnitude of intra-fraction patient movement in this study were similar to the findings given in other published studies. Linthout et al. (13) evaluated the mean intra-fraction patient movement for head and neck patient in lying posture and reported 0.0mm, 0.3mm, -0.5mm, -0.1°, 0.1° and -0.2° in the x, y, z, u, v, w directions, respectively. Pang et al. (11) showed that the mean translational intra-fraction patient movement in all directions ranged from -1.8 to 1.1 mm and that the calculated mean overall intra-fraction patient movement was 0.3mm for head and neck patients in lying posture. For treatments in the upright posture, McCarroll et al. (16) reported a range of the order of several millimeters for intra-fraction patient movements. Balakin et al. (17) reported up to a 3 to 4mm shift in the thermoplastic mask during proton beam radiation therapy while in the sitting posture. In our study, the mean intra-fraction patient movements were -0.08mm, 0.71mm, -0.52mm, 0.10°, 0.09°, and -0.04° in the x, y, z, u, v, w directions, respectively, and only the intra-fraction patient movements in the y and z direction exceeded 0.5mm. The aforementioned intra-fraction patient movement was based on 30-minute (SD 7 minutes) treatment time. Although 6DTC could offer wide range of beam entry angles, its true advantage is in the selection optimal beam entry angles rather than adding more beams to a plan. Two to three beam entry angles can often achieve an optimal plan in particle radiotherapy. Adding more beam entry angles will prolong the treatment time and cause a larger intra-fraction patient movement with minimal gain in improving the dose distribution.

There are several factors that contribute to intra-fraction patient movement. Strategies devised to mitigate each specific cause could potentially reduce the associated errors. From our experience, when patients sit in a chair for a long time, they tend to sag their heads from fatigue, although being immobilized in the thermoplastic head mask. Tilting the chair slightly backward might overcome this movement. A few patients complained about the mask being too tight for the jaw, making them uncomfortable. Pitching the chair five to ten degrees backwards during the planning stage would make the patient lean against the head/shoulder fixation interface plate and could reduce the discomfort. Pitching the chair, however, requires a significant translation of the chair rotating platform between beam applications to perform isocentric treatments which could induce additional errors. In general, finding a comfortable yet secure position should relieve stress and reduce the intra-fraction patient movement.

## Conclusions

In this study, the feasibility for ion beam radiotherapy using the 6DTC in upright seated posture and the performance stability of the 6DTC were demonstrated. Over the span of 150 fractions of treatment (nine months), our results indicated that the position accuracy and intra-fraction patient movement in upright seated posture were similar to published data for lying posture, which is considered acceptable by the state of the art of current clinical practice. Due to the inevitably increased physical demand on patients in sitting posture, however, an improved immobilization method to further reduce intra-fraction patient movement is desirable.

## Data Availability Statement

The raw data supporting the conclusions of this article will be made available by the authors, without undue reservation.

## Ethics Statement

The studies involving human participants were reviewed and approved by the Institutional Review Board of Shanghai Proton and Heavy Ion Center. The patients/participants provided their written informed consent to participate in this study. Written informed consent was obtained from the individual(s) for the publication of any potentially identifiable images or data included in this article.

## Author Contributions

JS, XW and YS contributed conception and design of this study. JS and YS organized the database. JS and ZC performed the statistical analysis. JS wrote the first draft of the manuscript. LK, DY, JM, XG, XW and YS wrote sections of the manuscript. All authors contributed to the article and approved the submitted version.

## Funding

This project was sponsored by Science and Technology Development Fund of Shanghai Pudong New Area (Project No. PKJ2019-Y08), Shanghai Academic/Technology Research Leader (Project No. 18XD1423000) and Science and Technology Commission of Shanghai Municipality No. 15411950100.

## Conflict of Interest

The authors declare that the research was conducted in the absence of any commercial or financial relationships that could be construed as a potential conflict of interest.
